# Heterogeneity in network structure switches the dominant transmission mode of infectious diseases

**DOI:** 10.1093/pnasnexus/pgad227

**Published:** 2023-07-11

**Authors:** Pratyush K Kollepara, Rebecca H Chisholm, Joel C Miller

**Affiliations:** Department of Mathematical and Physical Sciences, La Trobe University, Plenty Rd and Kingsbury Dr, Melbourne, 3086 VIC, Australia; Department of Mathematical and Physical Sciences, La Trobe University, Plenty Rd and Kingsbury Dr, Melbourne, 3086 VIC, Australia; Melbourne School of Population and Global Health, The University of Melbourne, Grattan St, Melbourne, 3010 VIC, Australia; Department of Mathematical and Physical Sciences, La Trobe University, Plenty Rd and Kingsbury Dr, Melbourne, 3086 VIC, Australia

**Keywords:** public health intervention, multiple transmission routes, sexually transmitted infection, heterogeneous transmission

## Abstract

Several recent emerging diseases have exhibited both sexual and nonsexual transmission modes (Ebola, Zika, and mpox). In the recent mpox outbreaks, transmission through sexual contacts appears to be the dominant mode of transmission. Motivated by this, we use an SIR-like model to argue that an initially dominant sexual transmission mode can be overtaken by casual transmission at later stages, even if the basic casual reproduction number is less than one. Our results highlight the risk of intervention designs which are informed only by the early dynamics of the disease.

**Significance Statement**

This article explores the risk from secondary transmission routes of diseases which spread through sexual contact. This is important because recently emerging infectious diseases such as Ebola, Zika, and mpox spread through both sexual transmission and other modes of transmission. Our results suggest that a secondary transmission route—which is not dominant in the initial stages—can significantly alter the course of the epidemic and lead to more infections than expected in the later stages of the epidemic.

## Introduction

The risk of emerging diseases continues to grow due to increases in human mobility and human–animal interactions. In just 20 years, we have seen multi-country epidemics following the emergence of SARS-CoV-1, H1N1 influenza (swine flu), SARS-CoV-2, Ebola, and mpox (also known as monkeypox) into human populations. Additionally, we have seen Zika, Cholera, drug-resistant Malaria, and other diseases spread from one human population into other previously unexposed populations, causing significant harm to public health.

Mpox is a neglected tropical disease, endemic in Western and Central Africa, which frequently spills over from animal reservoirs into human populations. It has been documented to spread via respiratory droplets and skin-to-skin contact, which we refer to as ‘casual contact’ in this article. The basic reproduction number for casual contact has been observed to be less than one (1–4). In the recent outbreaks of this disease in nonendemic regions (mainly in the Americas and Europe), sexual contact was identified as the dominant cause of transmission with a reproduction number greater than one (5, 6).

**Fig. 1.**
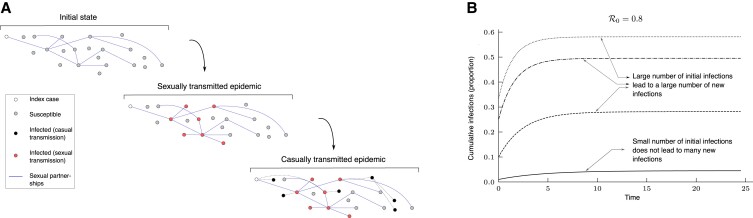
This figure illustrates the hypothesis that we explore in this article. The ‘sexual’ epidemic (with a reproduction number greater than one) causes many infections in the population, each of which acts as the initial seed for the ‘casual’ epidemic (with reproduction number less than one), causing a significant number of infections. A) The solid edges show the sexual partnership network that exists among the nodes. Casual transmission is homogeneous (equivalent a fully connected network), so the casual partnership network is not shown. The initial state shows the population in which an infection is introduced (the index case). The ‘sexually transmitted epidemic’ state shows the disease transmitted along some (but not all) edges of the sexual partnership network. The infections caused during the sexual epidemic act as a seeds for the ‘casually transmitted epidemic’. Black dashed edges show casual transmission events (for illustrative purposes, we ignore sexual transmissions seeded from the ‘casual epidemic’). On average, an infected node leads to less than one direct casually transmitted infection, but the cumulative average number of descendants can be large. Further as there are many infected seeds from sexual transmission, the casually transmitted infections add up to a significant proportion of the population. B) Time series of cumulative infections in a standard SIR model with $R_{0}=0.8$. This shows that if the initial number of infections is not small, a significant number of new infections can occur even if the reproduction number is less than one i.e. even if individuals on average cause less than one new infection.

Due to the highly heterogeneous contact structure of sexual contact networks, a sexually transmitted infection where clearance of infection confers permanent immunity, in the absence of new susceptibles (due to lack of demographic dynamics), would be expected to have a small final size compared to a nonsexually transmitted disease with the same basic reproduction number (7–9). However, if there is an additional transmission mechanism with a reproduction number less than one (R<1) (1, 3), we would expect that each individual infected through sexual contact would seed an outbreak through this additional mechanism which (on average) would have R/(1−R) infections (subject to assuming the outbreaks are independent which would not be the case if the number of sexual transmissions makes up a sizable proportion of the population). If R is close to 1, the expected size of the outbreaks can be quite large. This raises concerns that an epidemic might be initially dominated by transmission through sexual contacts, but ultimately a large fraction (even the majority) of infections could occur through casual transmission (see Fig. 1). In this article, we explore the dynamics of such a disease, in which sexual transmission dominates at the start of the epidemic. We use a mathematical model to study the interplay between the transmission routes and analyze how their behavior changes.

Mpox is not the only disease with multiple transmission routes. Sexual transmission has been identified as a secondary transmission route in Ebola and Zika in addition to their primary transmission route (10–15). SARS-Cov-2 is primarily airborne but also spreads through contact with surfaces (16). Chagas disease, a neglected tropical disease, is primarily vector borne but also spreads through blood transfusions and oral routes (17, 18). Scabies can spread via skin-to-skin contact and through fomites (19). Trachoma spreads via close contact and is also vector borne (20).

This underlines the need for modeling of potential emerging diseases which have a casual contact reproduction number close to one and a sexual reproduction number greater than one. It would be expected that if the sexual transmission mode dominates at the start of the epidemic, intervention policies may focus only on sexual transmission and ignore transmissions from casual contacts. Despite the documentation of multiple transmission routes in several diseases, modeling literature on multiple transmission routes is sparse (21–25). Models similar to the ones presented here can also be found in models of sexual transmission for Zika virus (26, 27) and in percolation of clustered networks (28). In this work, we adapt the framework developed in ref. (23) to develop an SIR model that uses two routes of transmission: homogeneous mass action transmission (representing casual transmission) and a network with a heterogeneous degree distribution (representing sexual transmission).

## Methods

### Compartmental model

We build a model following an existing framework for sexual transmission and nonsexual transmission (23). For sexual transmission, we use a heterogeneous mean-field (annealed) network. The annealed network assumption means an individual changes their contacts on a time scale that is faster than transmission but maintains the number of contacts at a constant value. This assumption will be used to derive the reproduction number in the next subsection. For casual transmission, we assume that individuals mix homogeneously. At any time, all individuals are at identical risk of acquiring infection through casual transmission.

Starting with equations for the proportion of individuals in each disease stage and stratified by their number of sexual contacts (degree), the model can be reduced to a system of four differential equations. We denote the proportion of the total population which has *k* sexual contacts and with state *X* by $X_{k}$, where X∈{S,I,R}. The proportion of the total population with state $X=\sum X_{k}$ (summed over all degrees) is denoted by *X*. The proportion of the total population which has *k* sexual contacts is denoted as $N_{k}=S_{k}+I_{k}+R_{k}$. The per-partnership sexual contact transmission rate is $\beta_{1}$, the overall casual contact transmission rate is $\beta_{2}$ and the recovery rate is γ. Our governing equations are


(1) $$\;\frac{dS_{k}}{dt}=-\beta_{1}kS_{k}\pi_{I}-\beta_{2}S_{k}I$$

(2) $$\frac{dI_{k}}{dt}=\beta_{1}kS_{k}\pi_{I}+\beta_{2}S_{k}I-\gamma I_{k}$$

(3) $$\;\frac{dR_{k}}{dt}=\gamma I_{k},$$

where $\pi_{X}\equiv \frac{\sum kX_{k}}{\sum kN_{k}}$ is the probability that a random sexual contact occurs with an individual in the state *X*. We can also define the probability generating function


(4) $$\Psi (x)=\sum N_{k}x^{k}.$$

It can be immediately seen that $\Psi^{\prime }(1)=\sum kN_{k}=\langle K\rangle$ and $\Psi^{\prime \prime }(1)=\langle K(K-1)\rangle$. Using the differential equation for $S_{k}$


(5) $$0=\frac{dS_{k}}{dt}+(\beta_{1}k\pi_{I}+\beta_{2}I)S_{k},$$

(6) $$\Rightarrow S_{k}=S_{k}(0)e^{-k\int_{0}^{t}\;\beta_{1}\;\pi_{I}\;d\tau }e^{\;-\;\int_{0}^{t}\;\beta_{2}I\;d\tau }.$$

Let


(7) $$\theta (t)\equiv e^{-\int_{0}^{t}\;\beta_{1}\;\pi_{I}\;d\tau },$$

for which


(8) $$\frac{d\theta }{dt}=-\beta_{1}\pi_{I}\theta .$$

Similarly, let


(9) $$\chi (t)\equiv \;\int_{0}^{t}\beta_{2}I\;d\tau ,$$

(10) $$\Rightarrow S_{k}(t)=S_{k}(0)\theta^{k}e^{-\chi }.$$

If we assume that only sexual transmission is present, then $\theta {\left(t\right)}^{k}$ is the proportion of degree *k* individuals that are susceptible at time *t*. This proportion equals the probability that a randomly chosen individual of degree *k* is not infected, or in other words, was never exposed to transmission up to that time. Similarly, if only casual transmission is present, then $e^{-\chi (t)}$ is the probability of never being exposed to transmission up to time *t*. When both transmission modes are present, the probability that a randomly selected individual is not infected is equal to the product of the probability that the individual was never exposed to sexual transmission from an infected individual and the probability that the individual was never exposed to casual transmission from an infected individual. Therefore, $\theta^{k}$ and $e^{-\chi }$ can be interpreted as the probability of not being exposed to sexual transmission (for the given degree *k*) and casual transmission, respectively. For the initial conditions, we assume that the proportion of degree *k* individuals initially infected is $\rho_{k}$. We can now find an expression for S(t)


(11) $$S_{k}(0)=(1-\rho_{k})N_{k},$$

(12) $$\;\Rightarrow S(t)=\sum S_{k}(t)=e^{-\chi }\sum (1-\rho_{k})N_{k}\theta^{k}.$$

Similarly,


(13) $$\pi_{S}=\frac{\sum kS_{k}}{\Psi^{\prime }(1)}=\frac{e^{-\chi }}{\Psi^{\prime }(1)}\sum (1-\rho_{k})kN_{k}\theta^{k}.$$

The infinite system of Eqs. 1, 2, and 3 can then be reduced to four differential equations


(14) $$\;\frac{dR}{dt}=\gamma I=\gamma (1-S-R),$$

(15) $$\frac{d\pi_{R}}{dt}=\gamma \pi_{I}=\gamma (1-\pi_{S}-\pi_{R}),$$

(16) $$\;\frac{d\theta }{dt}=-\beta_{1}(1-\pi_{S}-\pi_{R})\theta ,$$

(17) $$\;\frac{d\chi }{dt}=\beta_{2}(1-S-R).$$

**Fig. 2.**
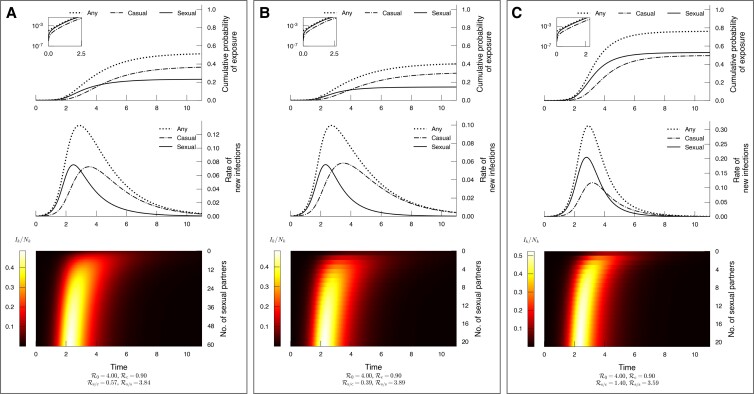
The probability of exposure through casual transmission may exceed that of sexual transmission even when the reproduction number for casual transmission is less than one ($R_{c}<1<R_{s/s}$). The parameters for each panel are, A) $N_{0}=0.34,\;k_{max}=60,\;\alpha =2$; B) $N_{0}=0.75,\;k_{max}=20,\;\alpha =2$; C) $N_{0}=0.34,\;k_{max}=20,\;\alpha =3$. The recovery rate is γ=1 for all panels. The reproduction numbers shown at the bottom of each panel are—$R_{c}$: casually transmitted infections caused by any individual, $R_{s/s}$: sexually transmitted infections caused by a sexually infected individual, $R_{s/c}$: sexually transmitted infections caused by a casually infected individual. We use $R_{0}=4$ and $R_{c}=0.9$ and the rest of the reproduction numbers are determined. All the plots in the figure are time-series. In each panel, the first plot shows the probability that a randomly selected individual (i) was exposed to any transmissions, given by (1−S), (ii) was exposed to a sexual transmission event, given by 1−Ψ(θ) and shown by solid line, (iii) was exposed to casual transmission, given by $1-e^{-\chi }$ and shown by dot-dashed line. The second plot shows the rate at which new infections are created by (i) any transmission route, (ii) sexual transmission, (iii) casual transmission. The third plot shows the density of active infections ($I_{k}/N_{k}$) in each group (stratified by the number of contacts *k*). In panels A) and B), the first plots show that the probability of exposure to sexual transmission is initially high but later in time, casual transmission starts to dominate.

These equations can be solved numerically, using Eqs. 12 and 13. In the limit $\rho_{k}\to 0$, $S(t)=\Psi (\theta )e^{-\chi }$. We can interpret Ψ(θ) as the probability of not having been exposed to sexual transmission, and $e^{-\chi }$ as the probability of not having been exposed to transmission through the casual contact route. In Fig. 2, we plot 1−Ψ(θ) and $1-e^{-\chi }$ as a function of time. These are the probabilities that a randomly chosen individual was exposed to sexual transmission at least once, or to casual transmission at least once, respectively. In solving the system of differential equations (14–17) numerically, we use $\rho_{k}\propto k$ as the initial conditions where all the $\rho_{k}\ll 1$. This initial condition is used because we assume that an individual with more sexual contacts is more likely to get infected.

### Sexual transmission network

For the sexual transmission network, we use a class of networks whose degree distribution P(K=k) (we use $N_{k}$ as a shorthand for this) is given by


(18) $$P(K=k)=N_{k}=\left\{ \begin{array}{cc} N_{0} & \text{ for }k=0\\ Ck^{-\alpha } & \text{ for }k\geq 1\;\&\;\;k\leq k_{max}\\ 0 & \text{ for }k>k_{max} \end{array}\right.$$

for integer values of *k*, where $N_{0}$ is proportion of individuals in the population that are sexually inactive and can be chosen arbitrarily, $C=\frac{1-N_{0}}{\sum k^{-\alpha }}$ is a normalization constant and $k_{max}$ is the maximum number of sexual contacts an individual is allowed to have. The exponent −α with α>0 corresponds to decay at large *k*, but much slower than exponential. Often, scale-free networks or Erlang distributed networks are used for modeling of sexual transmission networks (29–31) to capture the slow decay at large *k*. Our network differs from these networks by including a cutoff on the degree and the presence of nodes with zero degree. In the next subsection, we show that the second moment of the distribution, $\left\langle K^{2}\right\rangle$, forms a component of $R_{0}$. The qualitative features of our results will persist irrespective of the details of the distribution. What is crucial is that the distribution has sufficient high-degree individuals that $\left\langle K^{2}\right\rangle$ is large compared to $\langle K\rangle^{2}$, and that the high-degree individuals comprise a sufficiently small proportion of the population.

### Next generation matrix and basic reproduction number

The reproduction number can be obtained by calculating the largest eigenvalue of the next generation matrix *G* (32, 33), whose elements are:


(19) $$G_{kl}=\frac{S_{k}}{\gamma }\left(\frac{k\ell \beta_{1}}{\Psi^{\prime }(1)}+\beta_{2}\right).$$

Another approach is by using a lower dimensional description of the system. At each generation, *g*, the newly infected population can be grouped into those who have been infected through sexual contact (with proportion $N_{s}^{g}$) and those who have been infected through casual contact (with proportion $N_{c}^{g}$). In the next generation, this grouping is transformed by a next generation matrix (34)


(20) $$\left[\begin{array}{c} N_{c}^{g+1}\\ N_{s}^{g+1} \end{array}\right]=\left[\begin{array}{cc} R_{c} & R_{c}\\ R_{s/c} & R_{s/s} \end{array}\right]\left[\begin{array}{c} N_{c}^{g}\\ N_{s}^{g} \end{array}\right].$$

The average number of new infections caused by an individual through casual transmission is $R_{c}$. From the model, we see that the transmission rate is $\beta_{2}$ for casual transmission and 1/γ is the time spent in the infectious stage, so $R_{c}=\frac{\beta_{2}}{\gamma }$. The average number of new sexually transmitted infections caused by an individual who was infected casually is $R_{s/c}=\frac{\beta_{1}\langle K\rangle }{\gamma }$. The average number of new sexually transmitted infections caused by an individual who was infected sexually is $R_{s/s}=\frac{\beta_{1}\langle K^{2}\rangle }{\gamma \langle K\rangle }$. These can be computed by considering the following.

There are two network layers, a homogeneous layer for casual contact and a sexual transmission layer with degree given by random variable *k* and a distribution $N_{k}$. This leads to four types of reproduction numbers:

1. Casual infections caused by a casually infected individual: the casual contact network distribution is homogeneous with all transmitting at rate $\beta_{2}$ with an average duration of 1/γ. So, the expected number of new casual infections per infected individual is $\frac{\beta_{2}\ell }{\gamma }$.
2. Casual infections caused by a sexually infected individual: the expected number of new casual infections is also $\frac{\beta_{2}}{\gamma }$, because being infected through sexual contact does not affect transmission through the contact network.
3. Sexual infections caused by a sexually infected individual: The expected number of infections caused by a node of degree *k* is $\frac{\beta_{1}k}{\gamma }$ and the probability that a newly infected node has degree *k* is $\frac{N_{k}k}{\sum N_{\ell }\ell }$ (the higher the degree, the higher is the chance of contacting an infected individual). Thus, the expected number of infections is proportional to $\frac{\beta_{1}\langle K^{2}\rangle }{\gamma \langle K\rangle }$.
4. Sexual infections caused by a casually infected individual: the number of infections caused by a node of degree *k* is $\frac{\beta_{1}k}{\gamma }$. The probability that the newly infected node has degree *k* is $N_{k}$, and the expected number of infections is $\frac{\beta_{1}}{\gamma }\langle K\rangle$.

Note that in items (1) and (2) in the list above, the reproduction numbers are identical, $R_{c}=\frac{\beta_{2}}{\gamma }$. For (3) and (4), using the appropriate normalization, $R_{s/s}$ and $R_{s/c}$, are obtained respectively (34). The largest eigenvalue of the matrix in Eq. 20 matches with that of the next generation matrix defined in Eq. 19.

### Final state relations

We can derive transcendental equations for the variables θ and χ at the end of the epidemic by assuming that the proportion of initial infections in the population is negligible, i.e. $\rho_{k}\to 0$ and R(0)→0. Starting with Eq. 17 and using Eq. 14 (35, 36),


(21) $$\chi (\infty )-\chi (0)\;=\int_{0}^{\infty }\beta_{2}I\;dt,$$

(22) $$\;\chi (\infty )=\;\frac{\beta_{2}}{\gamma }R(\infty ).$$

At the end of the epidemic, I(∞)=0, and S(∞) can be obtained using Eq. 12


(23) $$\chi (\infty )=\frac{\beta_{2}}{\gamma }(1-e^{-\chi (\infty )}\Psi (\theta (\infty ))).$$

The equation for θ can be derived by considering its definition and using Eq. 15


(24) $$\theta (\infty )=\exp\;\left(-\int_{0}^{\infty }\beta_{1}\pi_{I}\;dt\right)=\exp\;\left(-\frac{\beta_{1}}{\gamma }\pi_{R}(\infty )\right).$$

At the end of the epidemic, $\pi_{I}(\infty )=0$, and $\pi_{S}(\infty )$ can be obtained from Eq. 13


(25) $$\theta (\infty )=\exp\;\left(-\frac{\beta_{1}}{\gamma }\left(1-e^{-\chi (\infty )}\theta (\infty )\frac{\Psi^{\prime }(\theta (\infty ))}{\Psi^{\prime }(1)}\right)\right).$$

Thus, Eqs. 23 and 25 together are transcendental equations for the final state of χ and θ. We solve them numerically through recursion and use the solutions for constructing Fig. 4.

## Results

**Fig. 3.**
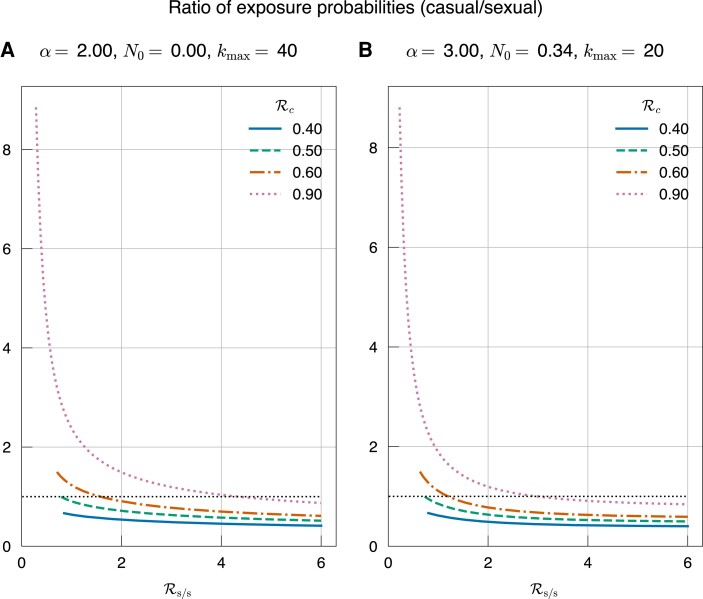
Ratio of exposure probabilities (casual w.r.t sexual) computed from simulations, for two different network structures and for values of $R_{c}=\{0.4,\;0.5,\;0.6,\;0.9\}$ (corresponding to the curves from bottom to top). From the simulations, the ratio of exposure probabilities (casual/sexual) at the end of the epidemic will match the expected number of descendants through casual transmission of each individual infected sexually. We can calculate this analytically under the assumption that the casual transmission chains do not intersect with other transmission chains (a reasonable assumption if the epidemic is small). Then, the casual transmission outbreak size is $R_{c}/(1-R_{c})$. So if $R_{c}<0.5$, casual transmission is not expected to dominate over sexual transmission because the outbreak size from one seed is less than one, and this can be seen in the figures. Furthermore, when the size of the sexual epidemic is very small, the ratio from the simulations will match the analytic prediction. This is also confirmed by the left end of the curves shown here. Note that the curves do not start from $R_{s/s}=0$, but rather from the value of $R_{s/s}$ for which $R_{0}=1$.

We are interested in studying if the presence of casual transmission (through homogeneous mass action) with a basic reproduction number $R_{c}$ less than one, in addition to sexual transmission (through a heterogeneous network) with a basic reproduction number $R_{s/s}$ greater than one, can affect the dynamics of an epidemic. We use a heuristic method to find some long-term behaviors of this problem. An infectious individual would on average create $R_{c}$ infections, which would lead to $R_{c}^{2}$ infections in the next generation, and so on, through casual contacts. For a large population, ignoring the fact that chains of transmission from multiple seeds may intersect and ignoring the sexual transmissions seeded by these chains, we can see that the total number of casually transmitted infections originating from an infected individual approaches the sum $R_{c}+R_{c}^{2}+R_{c}^{3}+\cdots =\frac{R_{c}}{1-R_{c}}$. As $R_{c}\to 1$, the sum diverges and we would expect a large number of infections from casual contact for each ‘seed’ infected through sexual transmission. Casual transmission by a single infectious individual may not amount to a large proportion of infections, but when a significant proportion of the population is infected through sexual transmission, all the infections caused by casual transmission will add up to a considerable proportion. If $R_{c}>1/2$, then we can expect more than one casual infection per seed. These analytical observations are verified in the simulations by computing the ratio of final exposure probabilities (casual relative to sexual). This ratio from the simulations matches the outbreak size per seed $\left(\frac{R_{c}}{1-R_{c}}\right)$) when $R_{s/s}$ is very small (Fig. 3).

**Fig. 4.**
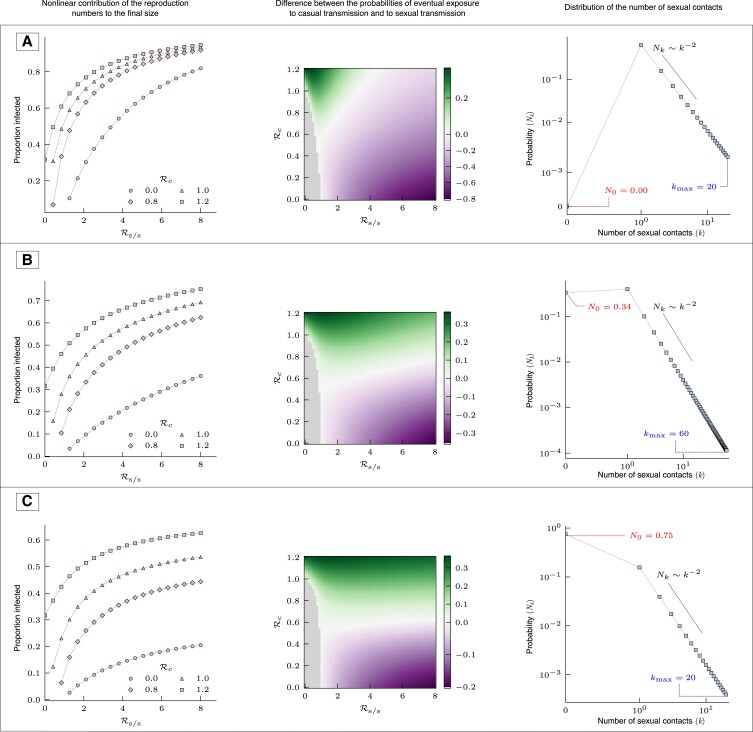
Sections of parameter space of the model. The first plot of each row shows how the final size or the proportion infected (R(∞)) in the epidemic, changes with $R_{s/s}$ for different values of $R_{c}$, the casual contact reproduction number. The second plot in each row shows a heat map of the difference between probability of exposure to casual transmission and probability of exposure to sexual transmission, at the end of the epidemic, plotted across the basic casual reproduction number and basic sexual–sexual reproduction number. The upper regions in the heatmap (top left in A) indicate the reproduction numbers for which casual transmission eventually dominates. The third plot shows the degree distribution and its parameters. For all the three cases, the recovery rate γ=1.

We study the same scenario using Eqs. 14–17 that incorporates casual transmission using a mass action type dynamics and sexual transmission using an annealed network chosen to produce a power law-like degree distribution, described in the Methods section. Our results show that the role played by the two transmission modes in the spreading of the disease at early times is not a good indicator of their roles at later times. The early dynamics are analyzed using the next generation matrix derived in Eq. 20. The stable distribution of transmissions, meaning the proportions of casual transmissions and sexual transmissions for each generation is obtained by the top eigenvector of the next generation matrix, $\left[\frac{R_{c}}{R_{0}},\;1-\frac{R_{c}}{R_{0}}\right]$. If $R_{c}\ll R_{s/s}$, then $R_{0}\approx R_{\text{s/s}}$ (23). Therefore, almost all of the early spread will be through sexual transmission.

In Fig. 2, we show the trajectory of the epidemic in three different ways, for three populations that have different sexual contact networks, but with $R_{c}=0.9$ and $R_{0}=4$. Since $R_{c}<R_{s/s}$, the probability of getting exposed to sexual transmission (1−Ψ(θ)) is initially higher than that of getting exposed to casual transmission ($1-e^{-\chi }$). However, as the epidemic progresses, the dominant mode of transmission may change and the probability of exposure to casual transmission may exceed that of sexual transmission. This behavior, which we refer to as ‘switching of the dominant transmission mode’, can occur over a wide range of parameter values, and the final size can be very sensitive to the presence of casual transmission (Fig. 4).

The mechanism for switching of the dominant transmission mode can be deduced from Eq. 12. The rate at which susceptibles deplete is higher for those with a larger degree. When the epidemic is starting, the first nodes to get infected sexually and recover are disproportionately those with the highest degree. Since the proportion of high-degree nodes is very small ($N_{k}∼k^{-\alpha }$), after the initial stage these nodes are depleted and the epidemic is sustained only through the more numerous but low-degree nodes which do not contribute much sexual transmission. Therefore, despite having a low basic reproduction number, the probability of exposure to casual transmission may be comparable to or even exceed the probability of exposure to sexual transmission as the epidemic progresses.

In contrast, if $R_{c}$ were larger than $R_{\text{s/s}}$, casual transmission would certainly dominate in the early times. However, as discussed above, sexual transmission events become less likely after a short period of time due to depletion of higher-degree susceptibles. So, casual transmission will dominate throughout the epidemic. Thus, the heterogeneous degree distribution (of the initially dominant mode) is playing an important role in switching the dominant transmission mode and the existence of two routes of transmission with disparate reproduction numbers is not sufficient for switching.

Fig. 2B shows that for the given population, about 15% of the population are exposed to sexual transmission, while about 30% are exposed to casual transmission by the end of the epidemic. Since the casual transmission epidemic depends on sexual transmission, interrupting all sexual transmission chains would reduce the overall probability of exposure to zero. Interrupting all casual transmission chains would take the overall probability of exposure to <15%. There will be a small number of sexual transmitted infections caused by casually infected individuals, but this is small compared to the purely sexual transmission chains as the probability of receiving sexual exposure has nearly reached its final value by the time casual transmission peaks. This is because the early phase of the epidemic depletes the high-degree susceptible population. For this population, the presence or absence of sexual transmission decides whether an epidemic is established or not. On the other hand, conditional on the establishment of an epidemic, casual transmission may be more influential in contributing to the final epidemic size. Fig. 4 shows that even if $R_{c}$ is smaller than one, reducing it to zero may lead to a larger reduction in final size than reducing $R_{\text{s/s}}$ by the same amount, depending on the characteristics of the sexual contact network and values of the reproduction numbers.

## Sensitivity analysis

The model explored in this work was inspired by Mpox, which has a sexual basic reproduction number which is greater than one and a casual basic reproduction number which is less than one. The parameters of our simulations are chosen in accordance with this. We found that there are regions in this parameter space in which the casual transmission route becomes dominant later in the epidemic.

Fig. 2 shows two parameter sets where we observe this change in the dominant transmission mode, and Fig. 4 shows the space of $R_{c}$ and $R_{s/s}$ for three sexual partnership networks, in which we observe the change in dominance of the transmission modes. This figure also shows the space where both $R_{c}$ and $R_{s/s}$ are less than one, but the total basic reproduction number is greater than one. We observe from the heat map in the first row that it is possible for either of the transmission routes to dominate.

In this section, we report the sensitivity analysis for the parameters of the model. Mainly, we are interested in the effect of the maximum number of partners in the sexual contact network on the results. The supplementary file shows heat plots (Figs. S1 and S2) for $R_{s/s}$, final probability of exposure to sexual transmission (1−Ψ(θ)) and the ratio of exposure probabilities of the two transmission routes ($\left(1-e^{-\chi })/(1-\Psi (\theta )\right)$). These heat plots show that the maximum allowed number of sexual partners ($k_{max}$) is very influential in determining $R_{s/s}$ but less so for the probabilities of exposure, in the range of values we have explored. Importantly, the change in the dominant transmission mode is largely determined by the values of the sexual transmission rate, $\beta_{1}$, and the casual basic reproduction number, $R_{c}$. These heat plots also show the parameter space where $R_{s/s}<1$ and $R_{c}<1$ but the combined basic reproduction number is greater than one, $R_{0}>1$, more clearly than Fig. 4. In that case, we see that casual transmission can be dominant even when $R_{c}<R_{s/s}<1$, for certain network parameters. The supplementary file also shows the time-series for three of these parameter sets. Switching of the dominant transmission route is not observed in these plots, while network structure does play a role in determining which route dominates (Figs. S3, S4 and S5).

## Discussion

In this article, we have presented and analyzed a model of a disease with two transmission routes: casual and sexual, based on ref. (23). We model casual transmission using homogeneous mass action dynamics and sexual transmission using an annealed network with heterogeneous degree distribution. The degree distributions from empirically observed sexual transmission networks are often described as long-tailed distributions ($P(X=x)∼x^{-\alpha }$ with X>0) (29, 31). However, the social and biological constraints on an individual’s sexual activity would imply that there is an upper limit on the number of sexual contacts. To account for this, distributions which have an exponential cutoff for large degrees are often employed (29, 30). Instead of an exponential cutoff, we impose a cutoff on the maximum degree and also allow for a nonzero probability for individuals with zero sexual contacts. We have approximated casual transmission using homogeneous mass action dynamics i.e. all individuals are identical. Casual transmission, although heterogeneous, has not been reported to have long-tail properties (37, 38). Relative to the large dispersion in the distribution of sexual contacts, the dispersion in the casual contact distribution would be negligible, justifying the homogeneous assumption. We have omitted many details such as age structure, sex, and sexual orientation stratification, variation in transmission rates etc. While including them would have made the model more suitable for fitting to empirical data, it would have made the parameter space much larger. Our goal is to demonstrate the potential of dominant mode switching triggered by heterogeneity in the initially dominant mode, and including these details would have obscured the underlying mechanism.

Sexual transmission as an additional route of transmission was identified for both Zika and Ebola viruses (12–15). In the case of Zika virus, a disparity in the sexual transmission rates has been observed between men and women. Modeling of this observation has shown that it can lead to two different epidemic transitions on the network, one of which causes the disease to spread in the sub-networks of men who have sex with men, while a second transition causes these outbreaks to spillover into the heterosexual community (26, 27). There are qualitative similarities between these results and ours—with a high enough casual reproduction number (but less than one), we see the sexually transmitted epidemic spillover into the sexually inactive sub-population from our model. However, in addition to these similarities, our results further show that there is a temporal dimension to these spillover effects, which can have significant policy implications. These results, however, may extend beyond sexual transmission (24). What is crucial for the switching phenomena is that there is enough heterogeneity to cause a small epidemic in a sub-set of the population, and other mechanisms use this small epidemic as a seed to grow into the rest of the population.

Sexual transmission in mpox was a discovery from the recent outbreaks in nonendemic regions. From observations in endemic regions, it was believed to spread through casual contact and zoonotic transmissions. Sexual contact was not documented as a transmission route (2, 4–6). This work started out as an attempt to explore the potential impact of casual transmission in later stages of the emerging mpox epidemics, which were mainly driven by sexual contact transmission. But since the time of writing, the outbreaks have started to subside. At its peak in mid-August 2022, about a thousand cases of mpox were detected daily across the world. Heightened awareness of the disease, contact tracing, COVID-19-induced social distancing, and a very small basic casual reproduction number may explain why the outbreaks did not continue to grow through casual transmission.

Although this analysis was originally motivated by mpox, our results are applicable to any SIR-like disease with a dominant sexual mode of transmission (basic reproduction number greater than one) and a secondary casual mode of transmission (basic reproduction number less than one). The main insight from our model is that the highly heterogeneous structure of sexual contact network leads to a small epidemic through sexual transmission, but this epidemic creates numerous seeds for a casual transmission epidemic that may lead to a comparable or even larger number of new infections. Even those who are sexually inactive can be at risk of infection as the epidemic progresses.

In terms of designing intervention policies, recognizing the possibility of changing dominant transmission modes has important implications:

1. Expected outcome of interventions: Due to the heterogeneous degree distribution, the final size of a disease spreading on a sexual contact network is expected to be smaller than that in a homogeneous population, for the same value of $R_{0}$. Our results show that final size estimates based only on sexual transmission could be significant underestimates. Underestimating the final size in this manner could have a decisive impact on the performance of interventions. If the interventions are intensive and can completely eliminate sexual transmission at early stages of the epidemic, then the secondary route of casual transmission will not pose any significant risk. On the other hand, if the interventions are not able to eliminate, but only moderate sexual transmission, then casual transmission could become dominant at a later time leading to a significantly larger final size than expected. Thus, intervention policies for curtailing sexual transmission, based on the early observations of a disease may fail to meet the expected outcomes if an initially insignificant casual transmission route is ignored.
2. Risk factors for different groups: For sexually transmitted infections, children are generally considered to be not at risk because they are sexually inactive. The degree distribution we have used accounts for a sexually inactive component of the population and the results show that this group is also at risk once casual transmission becomes dominant.
3. A broader space of interventions: When $R_{s/s}\gg R_{c}$, sexual transmission forms the main component to the basic reproduction number and a reduction in $R_{s/s}$ will have an appreciable effect on reducing $R_{0}$. However, subject to the structure of sexual contacts, a reduction in $R_{s/s}$ and in $R_{c}$ by the same amount will have a starkly different effect on the final size of the epidemic. Even if the casual reproduction number is less than one, policymakers should consider the disproportionate contribution of casual transmission to the final size and implement interventions on casual transmission along with the interventions on sexual transmission. As there is a temporal dimension to the dominance of different transmission modes, interventions will need to account for this and be updated depending on which transmission mode is dominating.
4. Critical community size: Infections that induce immunity typically spread as an epidemic wave, and then infection counts crash for a long period until immunity wanes or new susceptibles join the at-risk population. In small or heterogeneous populations the disease is likely to go extinct during this inter-epidemic phase (39). The possibility of long chains of casual transmissions may help a disease to persist in smaller communities than would happen otherwise.
5. Data collection: If the disease is initially dominated by sexual transmission, it would be a natural to allocate resources only on collecting sexual transmission data during the early phases of an outbreak. Since casual transmission can become dominant at later stages, early studies should also collect data on alternate routes of transmission to estimate the reproduction number.

In conclusion, for a disease with dominant sexual transmission and a secondary casual transmission component at the beginning of the epidemic, the dominant transmission route can switch and by the end of the epidemic, casual transmission may have a far larger impact than what would be expected from only sexual transmission. Therefore, policymakers should consider interventions against casual transmission along with the conventional approaches to curtailing sexual transmission.

## Supplementary Material

pgad227_Supplementary_Data

## Data Availability

All computer programs used in this article are available at https://github.com/Joel-Miller-Lab/casual-sexual-transmission.

## Previous presentation

These results were previously presented at SPARK and SPECTRUM Annual Meeting, Cape Schanck, Victoria, Australia in February 2023.

## Preprints

A preprint of this article is published at https://doi.org/10.1101/2022.11.28.22282692.
