# Supplementary material for: Heterogeneity in network structure switches the dominant transmission mode of infectious diseases
Source: PNAS Nexus. 2023 Jul 11;2(8):pgad227. doi: 10.1093/pnasnexus/pgad227 (PMC10393287; doi:10.1093/pnasnexus/pgad227)
Supplement: pgad227_Supplementary_Data [file pgad227_supplementary_data.pdf]

Heterogeneity in network structure switches the dominant transmission mode of infectious diseases

Pratyush K. Kollepara [1], Rebecca H. Chisholm [1,2], and Joel C. Miller [1]
[1] *Department of Mathematical and Physical Sciences, La Trobe University, Melbourne, Australia and*
[2] *Melbourne School of Population and Global Health,*
The University of Melbourne, Melbourne, Australia

I. SUPPLEMENTARY FIGURES: HEAT PLOTS

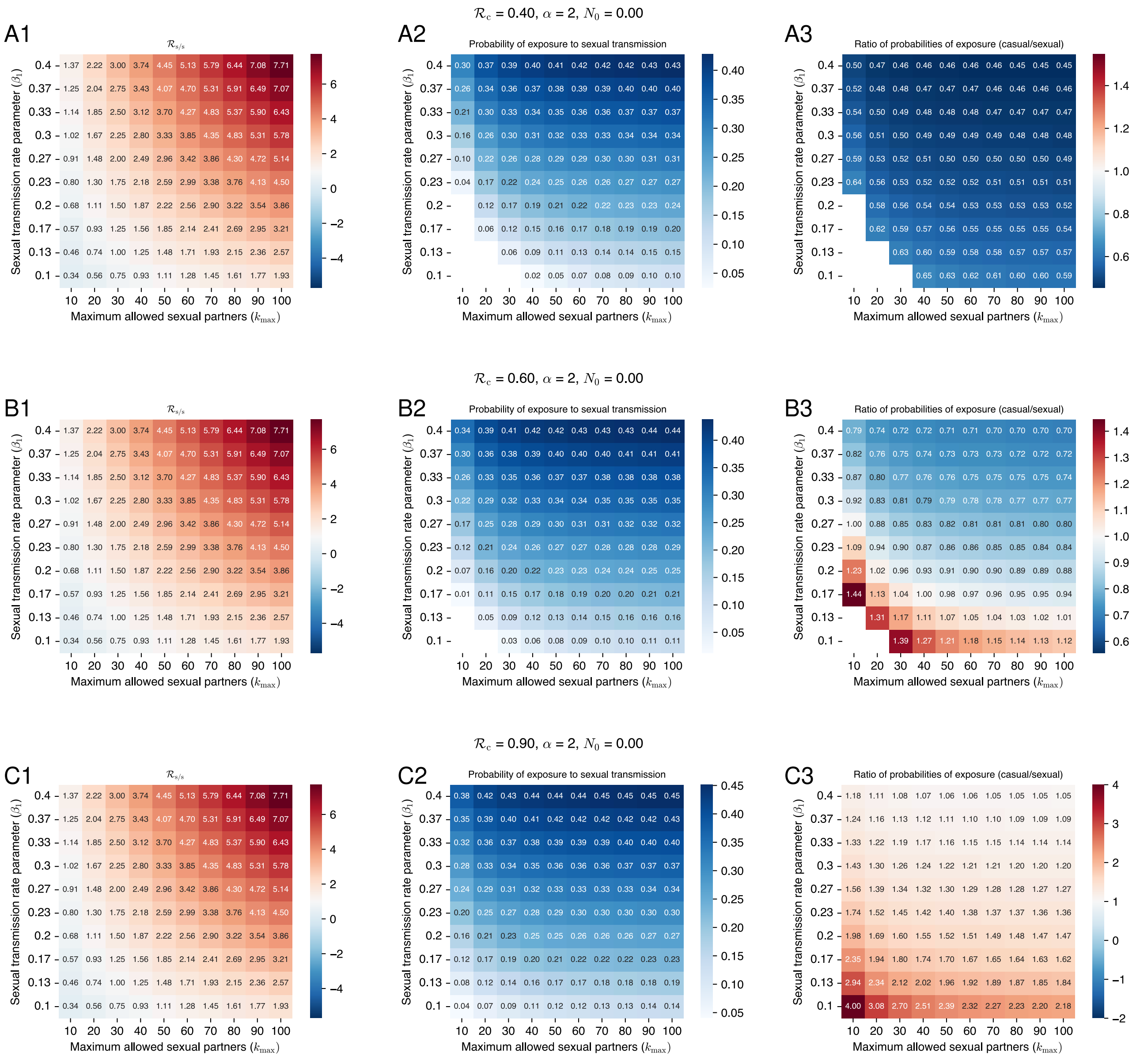

FIG. S1. For various values of the casual basic reproduction number, heatplots show the sexual-to-sexual basic reproduction number ($\mathcal{R}_{s/s}$), the final exposure probability for sexual transmission $(1-\Psi(\theta))$ and the ratio of final exposure probabilities (casual w.r.t sexual, $(1-e^{-\chi})/(1-\Psi(\theta))$, across the sexual transmission rate (β_1) and the maximum number of sexual partners allowed on a network $(k_{\max})$. The parameters are $\alpha = 2, N_0 = 0$.

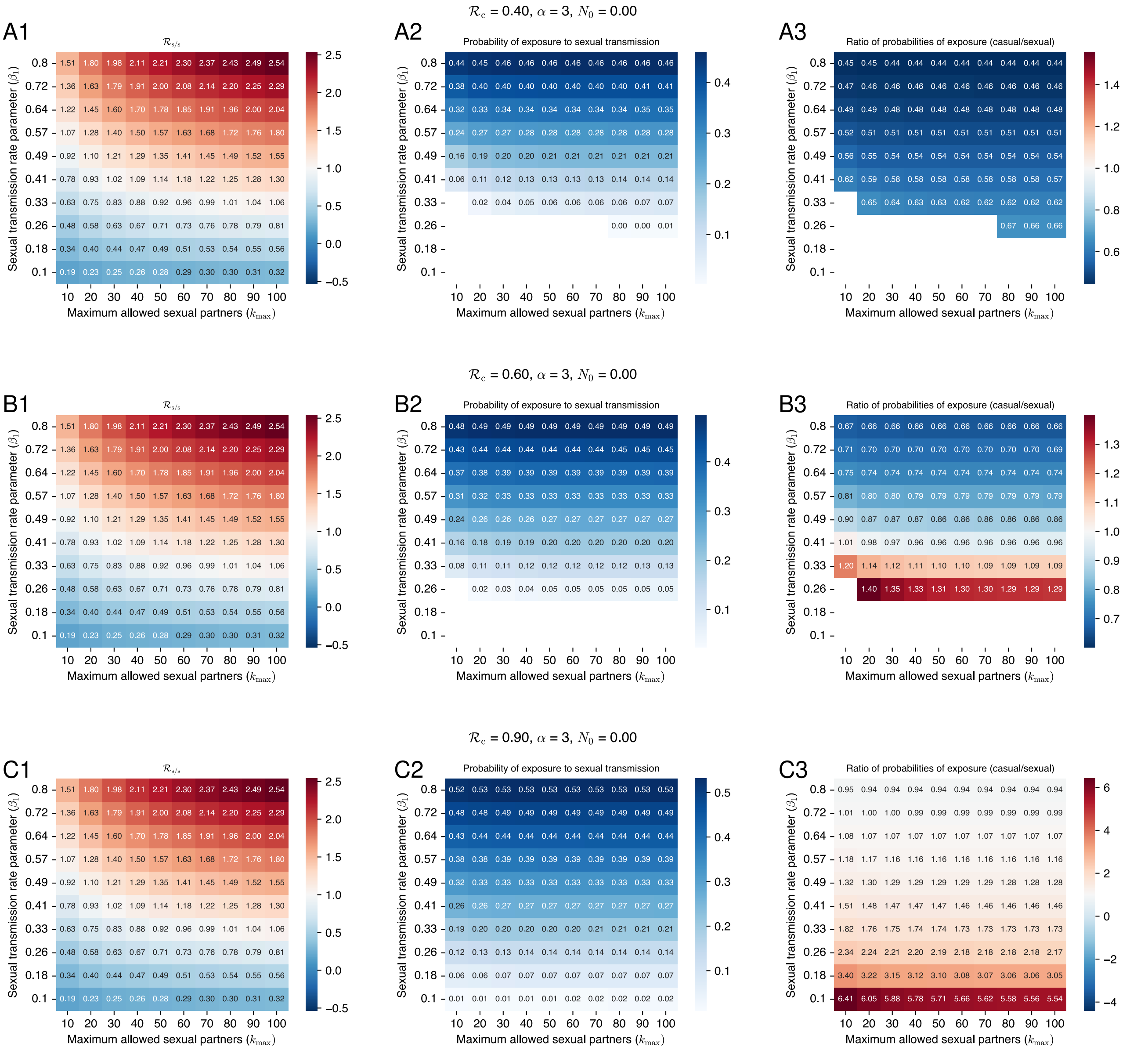

FIG. S2. For various values of the casual basic reproduction number, heatplots show the sexual-to-sexual basic reproduction number ($\mathcal{R}_{s/s}$), the final exposure probability for sexual transmission ($1 - \Psi(\theta)$) and the ratio of final exposure probabilities (casual w.r.t sexual, $(1-e^{-\chi})/(1-\Psi(\theta))$, across the sexual transmission rate (β_1) and the maximum number of sexual partners allowed on a network ($k_{\max}$). The parameters are $\alpha = 3, N_0 = 0$.

II. SUPPLEMENTARY FIGURES: TIMESERIES

Timeseries for some of the parameter values from the heat plots shown above.

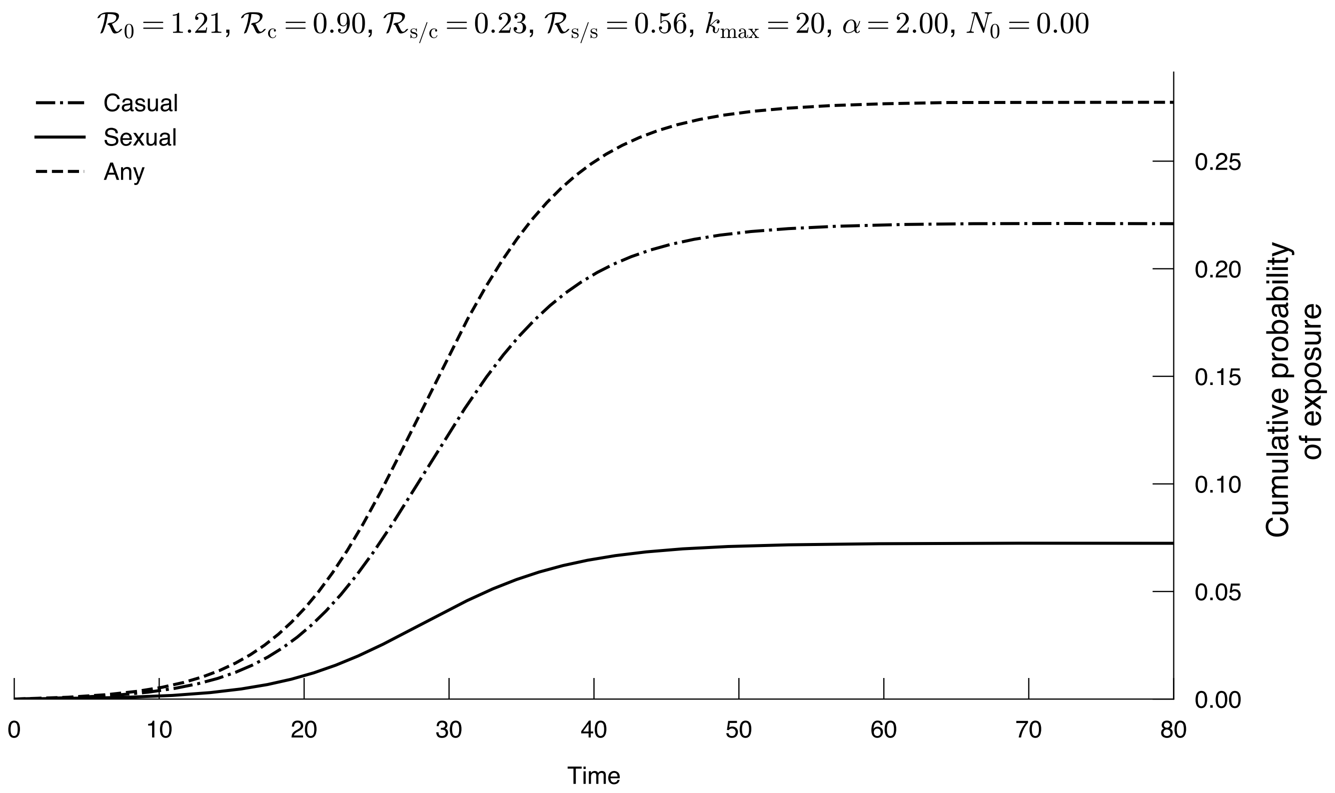

FIG. S3. Time series for $\mathcal{R}_{s/s} < \mathcal{R}_c < 1$ and $\mathcal{R}_0 > 1$.

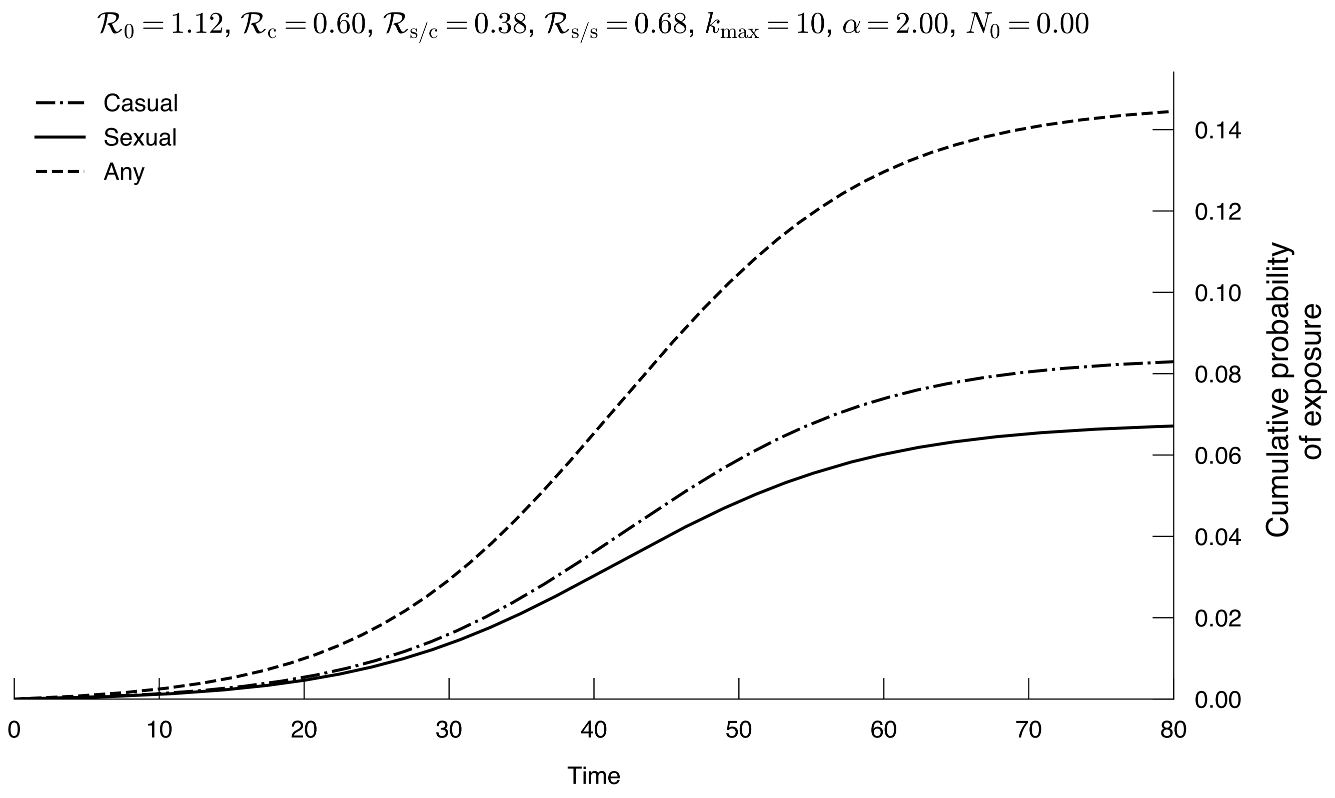

FIG. S4. Time series for $1 > \mathcal{R}_{s/s} > \mathcal{R}_c$ and $\mathcal{R}_0 > 1$. Casual transmission dominates.

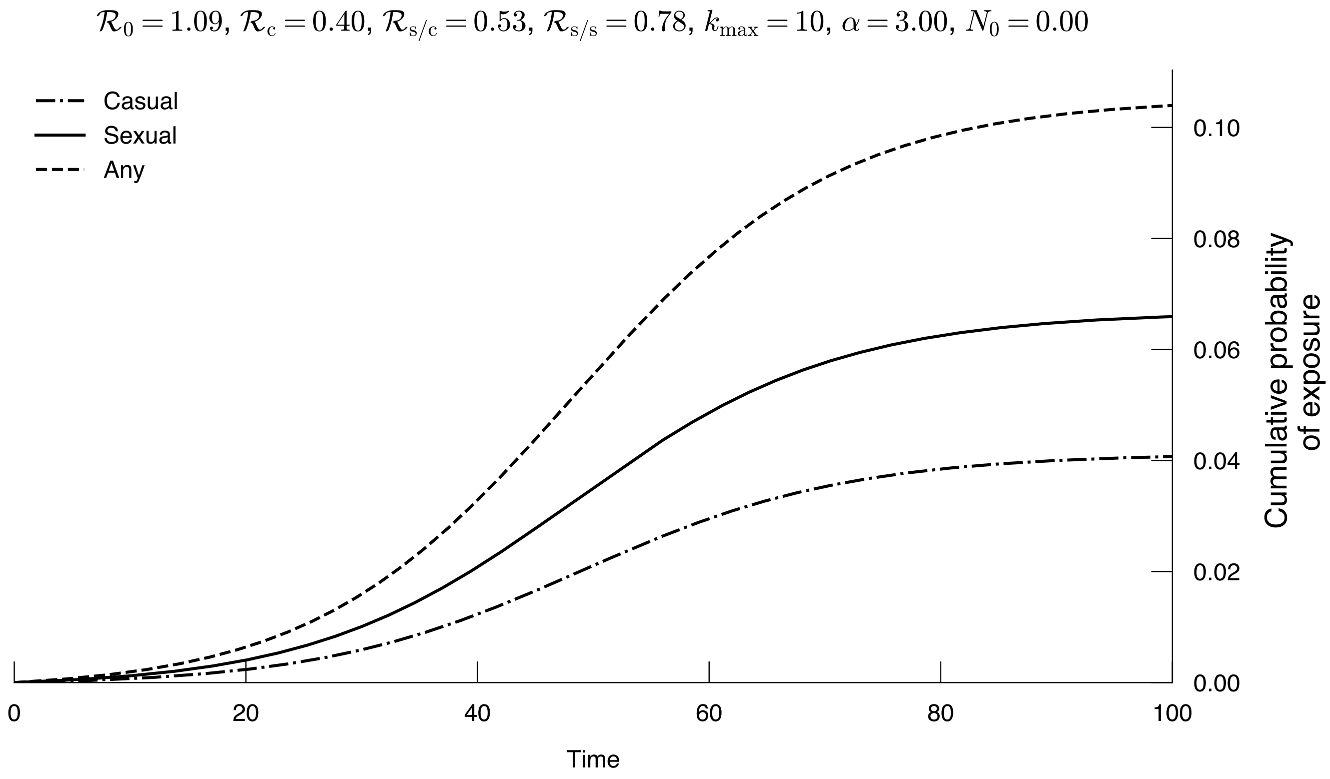

FIG. S5. Time series for $1 > \mathcal{R}_{s/s} > \mathcal{R}_c$ and $\mathcal{R}_0 > 1$. Sexual transmission dominates.
